# Hepatitis A surveillance: sensitivity of two information sources

**DOI:** 10.1186/s12879-018-3552-4

**Published:** 2018-12-07

**Authors:** Gloria Carmona, Marta Vilaró, Pilar Ciruela, Mireia Jané, Lluis Giralt, Laura Ruiz, Sergi Hernández, Àngela Domínguez, Working Group of the Epidemiological Surveillance Network of Catalonia, C. Arias, J. Álvarez, I. Barrabeig, N. Camps, M. Company, M. Carol, P. Godoy, A. Martínez, S. Minguell, I. Parron, M. R. Sala-Farré, J. Torres, A. Rovira, S. Manzanares, Working Group of the Microbiological Reporting System of Catalonia, F. Ballester, I. Pujol, M. Á. Benítez, A. Cebollero, J. Costa, J. Vila, A. Calderón, M. Curriu, M. Á. Domínguez, L. Calatayud, E. M. Dopico, M. J. Ferri, P. J. Ayala, J. Massa, D. Saenz, C. Gallés, P. Gassiot, F. Gómez, C. Molina, A. González-Cuevas, C. Guardia, M. Juanpere, M. Monsonis, C. Martí, N. Margall, L. Matas, M. Morta, S. Noguer, M. Olsina, P. Pérez, E. Padilla, M. Micó, M. O. Pérez-Moreno, T. Pumarola, F. Rodriguez-Frias, X. Raga, M. Ribelles, E. Sanfeliu, G. Sauc, G. Solé, A. Vilamala

**Affiliations:** 1 Public Health Agency of Catalonia (ASPCAT), Roc Boronat 81-95, 08005 Barcelona, Spain; 2 Departament de Medicina, Universitat de Barcelona, Barcelona, Spain; 3 CIBER Epidemiología y Salud Pública (CIBERESP), Barcelona, Spain

**Keywords:** Hepatitis A, Capture-recapture, Estimated incidence

## Abstract

**Background:**

The frequency of mild forms of hepatitis A, especially in children, could lead to underreporting. The objective of the study was to investigate the sensitivity of two surveillance systems, mandatory Statutory Disease Reports and the Microbiological Reporting System of Catalonia, using capture-recapture techniques.

**Methods:**

The study was conducted in Catalonia between 2011 and 2015. Hepatitis A cases reported to two independent surveillance systems were included: Statutory Disease Reports (SDR) and Microbiological Reporting System of Catalonia (MRS). The variables collected were: age, sex, year of declaration, size of municipality (< 10,000 and ≥ 10,000), country of birth (Spain or abroad), reporting centre (primary care/hospital) and notification method (electronic or paper). The capture-recapture analysis and the estimate of 95% confidence intervals were made using the Chapman formula for comparison of two sources, both for the estimate of the total number of cases and the stratification according to variables. Multinomial logistic regression was performed to obtain an adjusted estimate.

**Results:**

The SDR had a greater overall sensitivity than the MRS (48.8%; 43.5–55.6 vs. 19.3%; 17.2–21.9). In cases aged < 15 years the sensitivity of both systems was higher (76.6%; 72.7–81 vs. 25.2%; 20.9–29.5) than in cases aged > 15 years (25.5%; 22.8–28.3 vs. 12.1%; 10–14.2). For those born in Spain, the sensitivity was 57.2% (49.6–67.4) in the SDR and 27.1% (23.5–31.9) in the MRS, lower than that for foreign-born patients (58%; 51.2–66.8 vs. 49.1%; 43.4–56.6). In electronically-reported cases, the sensitivity was much higher in the SDR than in the MRS (47.2%; 42.3–52.1 vs. 9.4%; 6.5–12.3). No differences were observed according to sex, size of municipality, and year of declaration or reporting centre. The estimated total number of cases using the Chapman formula was very similar to the adjusted estimate (1121; 985–1258 vs. 1120; 876–1525), indicating the robustness of the results.

**Conclusions:**

The sensitivity of the SDR was greater than that of MRS, especially in patients aged < 15 years, although for patients born abroad the difference in sensitivity was lower. Reinforced surveillance combining the SDR and MRS improves the efficiency in the detection of cases.

## Background

The Statutory Disease Reporting System (SDR) is a passive surveillance system through which health professionals declare all infectious diseases subject to surveillance in Catalonia (Spain): the reporting systems and procedures are regulated by a Decree that must be complied with [1, 2]. Suspicion is sufficient to notify the SDR. The reporting physician may have the suspicion or confirmation of hepatitis A and, when reporting, should declare whether the notification is due to suspicion or laboratory confirmation.

The Microbiological Reporting System of Catalonia (MRS) is a surveillance system based on microbiologists reporting microorganisms that cause acute infectious diseases in Catalonia [3]. The MRS is based on reporting only laboratory-confirmed cases and does not notify cases ruled out (negative and false positive IgM).

The SDR and MRS are complementary and their integrated management constitutes a reinforced surveillance system capable of improving the detection of cases of diseases under surveillance.

Although all public or private health professionals are required to report any suspicion of any disease covered by the SDR, in practice there is underreporting by some professionals and, in consequence, the real incidence of the disease is underestimated. Among the main causes of underreporting are not knowing the obligation to report the disease, not appreciating the importance of doing so, and the pressures on health care [4].

Knowledge of the real incidence of diseases is also affected by underdetection. In the case of hepatitis A in children aged < 6 years, the infection is asymptomatic (without jaundice) in approximately 70% of children, which leads to underdetection. However, in adolescents and adults, 70% of cases are symptomatic [5]. The resources allocated by public health services to surveillance systems are limited, and periodic evaluation contributes to maximizing their efficiency [6].

The capture-recapture method is a statistical method for estimating the real incidence of diseases in a given population. It consists of studying, for two or more information sources, the number of cases detected by one source and the number of cases detected in the two or more sources used (coincident cases) to estimate cases not detected by the different sources used [7]. This method has the advantage of being much cheaper than others based on an active search for cases and allows similar results to be obtained [8]. The conditions for application of the capture-recapture methodology are [7, 8]: a) the population under study has to be closed, i.e., there should be no changes during the time in which the capture of cases occurs in the systems compared; b) there must be a method of determining whether an individual identified by one source is the same as an individual identified in the other system; c) each individual must have the same probability of being captured by either system; d) the systems must be independent. The aim of this study was to investigate the sensitivity of two surveillance systems using the capture-recapture method: the SDR and the MRS in Catalonia.

## Methods

### Information sources

The SDR is based on physicians reporting suspected cases of diseases deemed to be of mandatory report. One of the main functions of the SDR is epidemiological surveillance and control of these diseases and outbreaks of any aetiology considered as a priority for control in Catalonia, a Spanish region with 7.5 million inhabitants, which includes hepatitis A. The mere suspicion of hepatitis A by a physician means it must be reported as a suspected case. The reporting physician may have a suspicion or a confirmed case of hepatitis A and the report must state whether the case is suspected or laboratory confirmed (in which case the method of confirmation must be stated).

In Catalonia, there are definitions for suspected and confirmed cases of hepatitis A. Cases included in the SDR must meet one of the following definitions:

#### Suspected case

A case that meets the clinical case definition.

#### Clinical case definition

Person with discrete onset of symptoms (malaise, abdominal pain, anorexia, diarrhoea, nausea, intermittent vomiting, arthralgia) and one of the following three symptoms: fever, jaundice or elevated serum aminotransferase levels.

#### Confirmed case

(1) A case that meets the clinical case definition and is confirmed by laboratory tests: positive IgM against hepatitis A (anti-HAV positive), or (2) A case that meets the clinical case definition and is epidemiologically-linked to a confirmed case.

All cases reported to the SDR are reviewed to verify that they meet the definition of a suspected or confirmed case. Cases that do not meet the definition of suspected cases are classified as “non-cases” and are excluded.

This study only includes cases that met the case definition and were laboratory confirmed or were epidemiologically-linked to a confirmed case: suspected cases were excluded.

The MRS is a basic information system that belongs to the epidemiological surveillance network of Catalonia. The MRS collects information on microorganisms causing infectious diseases detected by laboratories participating in the system. The main objectives of the MRS are to provide information on certain diseases through the identification of the microorganisms involved and to determine trends and changes in the epidemiological patterns of microorganisms and microbiological resistance. Until 2015, the MRS was a voluntary surveillance system which covered 82% of acute hospital beds [2].

At the end of 2015, Catalonia drafted new legislation to harmonize the list of diseases to be subject to monitoring in accordance with the European norm. Under this new regulation, the MRS went from a voluntary reporting system to a system of obligatory declaration.

In the years included in the study (2011–2015), the laboratories participating in the MRS did so voluntarily. Professionals working in the Microbiology services of Catalonia involved in the system reported detections of specific anti-HAV IgM, providing data to identify the case (name).

Notification data in both the SDR and MRS are contained in an application in which data exploitation and analysis can be managed. The SDR and MRS are interconnected, since the two systems are complementary for communicable disease surveillance, but the independence of the sources is maintained. Cases that meet the clinical case definition and are epidemiologically linked to a confirmed case are not declared to the MRS because, by definition, they do not require confirmation, and are detected by the SDR.

### Data collection

MRS: We extracted all records coded for hepatitis A (confirmed cases) reported from January 2011 to December 2015 from the MRS. Likewise, we extracted all hepatitis records from the SDR dataset for the same study period. We then linked the databases using the personal identification code (PIC). When the PIC was not available, the date of report, age and sex were used to identify duplicates between the two sources. In cases with inconclusive/unclear matching, the hospital was used as a fifth matching criterion. The variables recorded for each case were age, sex, year of report, size of municipality (< 10,000 and ≥ 10,000), country of birth, type of report (electronic or paper) and centre of report (hospital or primary care centre).

SDR: Confirmed cases reported to the SDR according to the confirmed case definition were selected. Confirmed case: case that meets the clinical case definition and is laboratory-confirmed (specific anti-HAV IgM), or a case that meets the clinical case definition and is epidemiologically-linked to a confirmed case.

The study was not submitted for research ethics approval as the activities described were conducted as part of the legislated mandate of the Health Department of Catalonia, the competent authority for the surveillance of communicable diseases, which is officially authorized to receive, treat and temporarily store personal data on cases of infectious disease according to Decree 203/2015 of the 15 September which created the epidemiological surveillance network [2]. Therefore, all study activities formed part of public health surveillance and were thus exempt from institutional board review and did not require informed consent.

Personal data were used only for evaluation during the matching process. All the necessary measures to protect the confidentiality of personal data were taken during the whole evaluation (access to the data restricted to the personnel involved in data analysis and removal of personal data from the datasets after matching).

### Statistical methods

The total number of hepatitis A cases was estimated using the two-source capture-recapture method that uses Chapman’s formula [9] to reduce bias due to small samples:

$$ N=\frac{\left(L1+1\right)\left(L2+1\right)}{a+1}-1 $$

$$ 95\%\mathrm{CI}=\mathrm{N}\pm 1.96\sqrt{\frac{\left(\mathrm{L}1+1\right)\left(\mathrm{L}2+1\right)\left(\mathrm{L}1-\mathrm{a}\right)\left(\mathrm{L}2-\mathrm{a}\right)}{{\left(\mathrm{a}+1\right)}^2\left(\mathrm{a}+2\right)}} $$

where L1 is the number of cases in the SDR dataset, L2 is the number of cases reported to the MRS, and a is the number of cases captured by both systems. The sensitivity (Se) of case ascertainment by the two sources is calculated as the proportion of true cases detected by each source, i.e. Se(1) = L1/N for source 1 and Se(2) = L2/N for source 2. Sensitivity for the two sources when they are combined was calculated as the proportion of cases detected by one of the two sources or both, i.e. Se(1,2) = (L1 + L2-a)/N.

Estimates were made for the entire 5-year period and additionally stratified by age group, sex, year of report, size of municipality, country of birth, centre of report and type of report. (electronic or paper).

The independence of the sources was considered when applying the capture-recapture method [10, 11]. In the two-by-two table, where *a* represents cases reported by two sources or combinations of sources, *b* and *c* cases reported exclusively by either of the two sources, and *x* the estimated cases not reported by either of the sources, the odds ratio (OR = ax/bc) should not significantly differ from one.

A multinomial logit model [12, 13] was used to evaluate patient characteristics to the probability of capture, which allows more precise estimates of the number of estimated cases [14]. This identifies patient characteristics related to the probability of capture by the different sources. We used a backwards stepwise procedure (using likelihood ratio tests, with a *p*-value of > 0.2 as the criterion for removing variables from the model) [15, 16] to eliminate covariates, starting with a full model including all potential covariates and using the parameter estimates from the model to estimate the sizes of population subgroups and calculate the estimated incidence. We also derived confidence intervals which allow for the uncertainty in estimating the total number of cases. All analyses were made using R software version 3.0.1.

## Results

The distribution of patient characteristics by source is shown in Table 1. The mean age was 20.6 years in the SDR and 27.5 years in the MRS; 55% of patients were aged < 15 years in the SDR and 46% in the MRS. The male-female percentage was 56% vs.44% in the SDR, and 58% vs. 42% in the MRS. The number of Spanish-born patients was 34% in the SDR and 41% in the MRS and, in both cases was higher than the number of foreign-born patients. Paper was the main form of report (66% in the SDR and 83% in the MRS). The centre of report was 34% primary healthcare and 33% hospital in the SDR and 70% hospital reports and 30% primary healthcare in the MRS.

**Table 1:** Hepatitis A patient characteristics by source, Catalonia 2011–2015

	SDR (*n* = 547)	MRS (*n* = 216)
Age at report, years		
Mean (SD)	20.6 (18.6)	27.5 (23.1)
Median (IQR)	10 (29)	25 (40)
< 2 years, n (%)	29 (5.3%)	8 (3.7%)
2–4 years, n (%)	77 (14.1%)	26 (12.0%)
5–14 years, n (%)	196 (35.8%)	65 (30.1%)
15–24 years, n (%)	21 (3.8%)	8 (3.7%)
25–34 years, n (%)	92 (16.8%)	22 (10.2%)
35–44 years, n (%)	68 (12.4%)	28 (13.0%)
45–54 years, n (%)	33 (6.0%)	29 (13.4%)
> 55 years, n (%)	31 (5.7%)	29 (13.4%)
NAs	0 (0.0%)	1 (0.5%)
Sex, n (%)		
Male	308 (56.3%)	126 (58.3%)
Female	239 (43.7%)	90 (41.7%)
NAs	0 (0.0%)	0 (0.0%)
Year of report, n (%)		
Year 2011	134 (24.5%)	18 (8.3%)
Year 2012	127 (23.2%)	50 (23.1%)
Year 2013	107 (19.6%)	31 (14.3%)
Year 2014	101 (18.5%)	62 (28.7%)
Year 2015	74 (13.5%)	55 (25.5%)
NAs	4 (0.7%)	0 (0.0%)
Size of municipality, n (%)		
< 10,000 people	115 (21.0%)	75 (34.7%)
≥ 10,000 people	430 (78.6%)	127 (58.8%)
NAs	2 (0.4%)	14 (6.5%)
Country of birth, n (%)		
Spain	186 (34.0%)	88 (40.7%)
Other countries	92 (16.8%)	78 (36.1%)
NAs	269 (49.2%)	50 (23.1%)
Type of report, n (%)		
Electronic	184 (33.6%)	37 (17.1%)
Paper	363 (66.4%)	179 (82.9%)
NAs	0 (0.0%)	0 (0.0%)
Centre of report, n (%)		
Primary healthcare centre	184 (33.6%)	65 (30.1%)
Hospital	182 (33.3%)	151 (69.9%)
NAs	181 (33.1%)	0 (0.0%)

*NAs* Not available

### Capture-recapture analysis

The odds ratio (OR) to verify the independence of the two sources was 0.99 (95% CI 0.69–1.29), reinforcing the independence of the sources.

From 2011 to 2015, 547 cases were reported to the SDR (503 laboratory-confirmed and 44 without laboratory confirmation but epidemiologically-linked to a laboratory-confirmed case) and 216 to the MRS, 105 cases were included in both sources. The estimated total number of case reports expected during the whole period was 1121 (95% CI 985–1258) (Table 2). A decrease in the number of reported cases was observed between 2011 (134 cases) and 2015 (74 cases) and no outbreak was detected during the study period. The sensitivity was 48.8% (95%CI 43.5–55.6%) for the SDR and 19.3% (95%CI 17.2–21.9%) for the MRS (Table 3). The estimated total number of cases was statistically significant (*p*-value< 0.001) which means there were differences between the sensitivity of the two sources. Sensitivity increased to 58.7% (95%CI 54.9–62.4%) when the datasets were combined.

**Table 2:** Capture-recapture analysis of two datasets to estimate the total number of hepatitis A cases, Catalonia 2011–2015

		SDR		Total
		Identified	Not identified	
MRS	Identified	105	111	216
	Not identified	442	463	905
	Total	547	574	1121

*SDR* Statutory Disease Reporting

*MRS* Microbiological Reporting System of Catalonia

**Table 3:** Capture–recapture analysis of all hepatitis A cases and of subgroups, Catalonia 2011 to 2015

	No. records in SDR	No. records in MRS	Matched records	Calculated unreported cases	Estimated total no. of cases (95% CI)	SDR Sensitivity (%) (95% CI)	MRS Sensitivity (%) (95% CI)	Difference in sensitivity of the two sources	*p*-value
All cases	547	216	105	463	1121 (985–1258)	48.8 (43.5–55.6)	19.3 (17.2–21.9)	29.5%	< 0.001
Age at notification, years									
< 15 years	302	99	76	68	393 (357–430)	76.6 (72.7–81.0)	25.2 (20.9–29.5)	51.4%	< 0.001
> = 15 years	245	116	29	627	959 (687–1232)	25.5 (22.8–28.3)	12.1 (10.0–14.2)	13.4%	
Sex									0.667
Male	308	126	58	288	664 (554–775)	46.4 (39.8–55.7)	19.0 (16.3–22.8)	27.4%	
Female	239	90	47	172	454 (376–533)	52.6 (44.9–63.6)	19.8 (16.9–24.0)	32.8%	
Year of declaration									
Year 2011	134	18	9	155	268 (168–369)	50.0 (36.4–79.9)	6.7 (4.9–10.7)	43.3%	Ref
Year 2012	127	50	34	44	187 (158–216)	68.0 (58.8–80.6)	26.8 (23.2–31.7)	41.2%	< 0.001
Year 2013	107	31	21	41	158 (126–190)	67.7 (56.3–85.5)	19.6 (16.3–24.6)	48.1%	0.005
Year 2014	101	62	22	144	285 (207–364)	35.5 (27.8–48.9)	21.8 (17.1–30.1)	13.7%	0.015
Year 2015	74	55	19	104	214 (153–276)	34.6 (26.8–48.5)	25.7 (19.9–36.1)	8.9%	0.008
Size of municipality									0.070
< 10,000 people	115	75	33	102	259 (205–312)	44.5 (38.5–50.6)	29.0 (23.5–34.6)	15.5%	
> =10,000 people	430	127	72	270	755 (652–858)	57.0 (53.5–60.5)	16.8 (14.2–19.5)	40.2%	
Country of birth									< 0.001
Spain	186	88	50	101	325 (376–375)	57.2 (49.6–67.4)	27.1 (23.5–31.9)	30.1%	
Other countries	92	78	45	34	159 (138–180)	58.0 (51.2–66.8)	49.1 (43.4–56.6)	8.9%	
Type of notification									< 0.001
Electronic	184	37	17	186	390 (269–511)	47.2 (36.0–68.5)	9.5 (7.3–13.8)	37.8%	
Paper	363	179	88	281	735 (642–830)	49.4 (43.8–56.6)	24.4 (21.6–27.9)	15.8%	
Centre of notification									0.704
Primary health care centre	184	65	29	186	406 (310–503)	45.3 (36.6–59.5)	16.0 (12.9–21.0)	45.1%	
Hospital	182	151	76	103	360 (318–404)	50.5 (45.2–57.3)	41.9 (37.5–47.6)	5.6%	

Table 3 shows the unreported cases, the estimated number of cases and the sensitivity for both sources stratified by the characteristics considered.

**Fig. 1:**
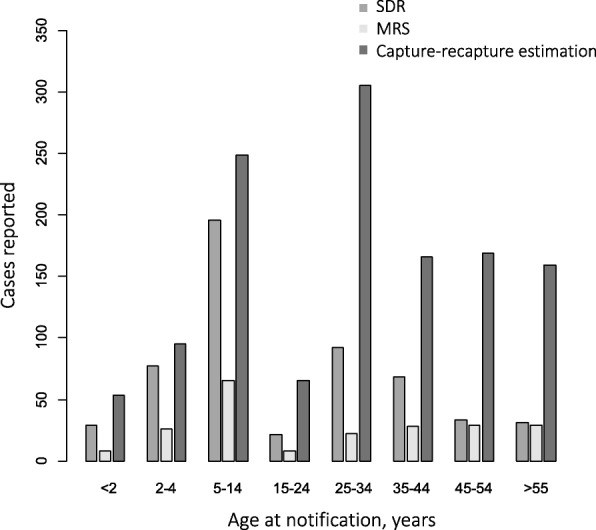
Cases by age groups reported to MRS and SDR systems and estimation cases by capture-recapture method

The sensitivity was 76.6% (95%CI 72.7–81.0) for the SDR and 25.2% (95%CI 20.9–29.5) for the MRS in the < 15 years age group, and 25.5% (95%CI 22.8–28.3) for the SDR and 12.1% (95%CI 10.0–14.2) for the MRS in the ≥15 years age group: the differences were statistically significant (*p*-value< 0.001). The 2–4 years age group had the highest sensitivity in both the SDR and MRS: 81.3% (95%CI 70.7–95.7) in the SDR and 27.5% (95%CI 23.8–32.3) in the MRS (Fig. 1). The sensitivity of the two sources was very similar between males (SDR 46.4%; 95%CI 39.8–55.7% and MRS 19.0%; 95%CI 16.3–22.8) and females (SDR 52.6%; 95%CI 44.9–63.6 and MRS 19.8 95%CI 16.9–24.0), without significant differences. No differences were observed according to the size of the municipality.

Comparing sensitivities by country of birth was statistically significant (*p*-value< 0.001) due mainly to the sensitivity of the MRS, with 27.1% (95%CI 23.5–31.9) for Spanish-born people and 49.1% (95%CI 43.4–56.6%) for foreign-born people. Paper reports were the most used by the two sources and the sensitivity for the SDR was similar, but electronic report had a sensitivity of 9.4% (95%CI 6.5–12.3%) while on paper it was 15.3% (95%CI 13.2–17.4%; *p* < 0.001). There were no significant differences in sensitivity according to the type of centre for either source: the sensitivity was 32.8% (95% CI 27.5–40.6%) in the SDR and 27.2% (95%CI 22.8–33.7) in the MRS for hospitals and 69.6 (95%CI61.2–80.6%) and 24.5% (21.6–28.5%) for primary healthcare.

The final multinomial logit model obtained after the stepwise procedure shows the characteristics that explain the identification of cases by the two sources and, therefore, the sensitivity of the two sources in identifying hepatitis A cases in Catalonia. The results of the multinomial logit model are shown in Table 4. In the final multivariate model, the variables considered statistically significant in defining the sensitivity of the two sources were the age at report (< 15 vs. > = 15 years), the country of birth and the type of report. Other variables that were also important in explaining the sensitivity of the two sources were the year of report (2011 to 2013 vs.2014 to 2015) and the centre of report. The odds of being identified by one of the two sources for the ≥15 years age group was 0.44 times (95%CI 0.26–0.74) that of the < 15 years age group; the odds for people born in Spain were 0.25 times (95% CI 0.15–0.43) that for foreign-born people; and the odds for paper reporting were 0.36 times (95%CI 0.21–0.64) that for electronic reporting. With these variables in the model, the adjusted estimate of the total number of cases was 1120 (95%CI 876–1525). This estimate was similar to that obtained by Chapman’s formula, suggesting the results were consistent and robust.

**Table 4:** Variables defining the sensitivity of the sources in detecting hepatitis A. Multinomial Logit model

	OR (95%CI)	*p*-value
Age at notification (≥ 15 years)	0.44 (0.26, 0.74)	0.002
Year of declaration (2014–2015)	0.65 (0.40, 1.07)	0.089
Country of birth (Spain)	0.25 (0.15, 0.43)	< 0.001
Type of notification (Paper)	0.36 (0.21, 0.64)	< 0.001
Centre of notification (Hospital)	1.59 (0.92, 2.76)	0.099

*OR* odds ratio

## Discussion

The results of this study provide robust estimates due to the fact that the sources studied are independent and because the assumption of independence was confirmed. Various authors have employed capture-recapture methods using independent sources that are the same or very similar to those we used to assess the sensitivity of different sources of the surveillance system for hepatitis A and other diseases. Overhage et al. [17] used two sources to assess hepatitis A surveillance completeness: a) automated electronic laboratory reports and b) spontaneous reporting by physicians: Durosoy et al. [18] also used two sources of hepatitis A surveillance: a) laboratory results and b) notifications by physicians. Matin et al. [19], in a study that assessed how many cases of hepatitis A are not reported, used three data sources: a) the Laboratory Reporting System, b) the Local Health Protection Unit and c) data derived from a specific project on hepatitis A genotyping.

The sensitivity of the SDR was greater than that of the MRS in both the global and the subgroup analyses. This may be because the SDR system is compulsory and has 100% coverage throughout Catalonia while, in the years analysed, the MRS system was voluntary and encompassed around 80% of Catalonia healthcare services. In addition, the SDR also includes a few clinically-compatible but not laboratory-confirmed cases which were epidemiologically-linked to a laboratory-confirmed case.

The new Decree [2] has established the obligatory nature of report to the MRS system by public and private hospital and primary healthcare microbiology laboratories in Catalonia. Future studies are required to determine whether this change affects the completeness of reporting from this source and, if so, to quantify the changes.

Other factors that contribute to the lower sensitivity of the MRS with respect to hepatitis A are that the serological tests necessary for the diagnosis of hepatitis A are not performed in all reporting microbiology laboratories, so some hospitals do not report cases. In addition, 70% of reports to the MRS are hospital-based and hepatitis A is detected mainly in primary healthcare centres.

Although the real incidence of clinical cases of hepatitis A is difficult to ascertain due to underreporting, evaluating the sensitivity of the sources used for disease surveillance is of interest because it may help to improve the detection of cases and the adoption of appropriate control measures [16, 20].

Our study underlines the importance of integrating information from different sources to monitor, prevent and control outbreaks [21].

Reviews of hepatitis A only or of foodborne diseases including hepatitis A in developed countries [22, 23] have found that hepatitis A is frequently underreported, as did the results of the study by Simmons et al. [24], who assessed the completeness of the notification system by comparing notifications with laboratory-confirmed cases of hepatitis A and other foodborne illnesses.

A meta-analysis of the completeness of the reporting of hepatitis A cases between 1997 and 2015 obtained heterogeneous results, ranging from 4 to 97%. Differences were attributed to factors such as the type of source used for case detection, reporting mechanisms (automatic methods, other methods) and staffing infrastructure [22].

A Turkish study by Durosoy et al. to determine the completeness of two sources (reporting and laboratory) for various diseases (hepatitis, brucellosis, syphilis, measles and HIV/AIDS) found that only 31.6% of cases of hepatitis A reported by the laboratory had been notified to the surveillance system. The incidence rates calculated from the cases reported to the surveillance system placed the region at the level of low incidence for hepatitis A, whereas if the cases identified by the laboratory were added, the incidence level increased and placed the region in the intermediate incidence zone [18]. A 2006 capture-recapture study conducted in England found high underreporting of hepatitis A cases, with a completeness of 27.8% (95% CI 19–38.7% [19].

In the present study, the sensitivity was significantly greater in cases in children aged < 15 years than in those aged ≥15 years, both for the SDR (76.6%, 72.7–81) and the MRS (25.2%, 20.9–29.5). The 2–4 years age group had the greatest sensitivity for both sources (81.3% for the SDR system versus 27.5% for the MRS). The sensitivity fell with increasing age, especially in the SDR, and from 45 years of age upwards was slightly below 20% for both sources. Although most capture-recapture studies do not analyse the sensitivity of the different sources across different age groups [23], the New Zealand study by Simmons et al. of foodborne diseases also showed a greater sensitivity for reporting hepatitis A in younger people [24]. It is difficult to find a simple explanation for the fact that more cases were reported in children than in adults, especially because hepatitis A is clinically more florid in adults. In any case, our results suggest the advisability of targeting health professionals to reinforce the reporting of cases in adults.

The sensitivity of the MRS was lower than that of the SDR throughout the study years, although it increased in more recent years, and was 25.7% (95% CI 19.9–36.1) in 2015. The improvement in the MRS results in recent years coincides with increased coverage of reporting to the MRS, especially in primary healthcare.

According to the country of birth, the notification of cases of hepatitis A in foreign-born people was more sensitive (*p* value < 0.001) than that in people born in Spain for the two sources. The country of origin is of great relevance for the surveillance of communicable diseases [25], and our results suggest that there is a greater concern on the part of respondents to report cases occurring in foreign-born people than those occurring in the native population. It may be that health professionals have a greater suspicion of hepatitis A in people born in countries where the disease is endemic than in people born in Spain which, in recent years, has seen a significant decrease in the disease incidence.

Although there were no significant differences in the results obtained in the two sources according to the type of centre, the SDR had greater sensitivity in cases reported by primary healthcare centres (69.6%) than in cases reported by hospitals (32.8%). In contrast, in the MRS, the sensitivity was slightly higher in hospitals (27.2%) than in primary healthcare centres (24.5%). The SDR results may be due to the fact that a percentage of hepatitis A cases, especially in children, do not present complications, and therefore are not hospitalized, with primary healthcare professionals detecting and reporting the disease.

In our study, the sensitivity of electronic reporting was higher than that obtained by paper-based reports for the SDR (47.2% vs. 31.1%), whereas the comparable figures for the MRS were 9.4% vs. 15.3%, with the differences being statistically significant. The greater implementation of electronic reporting in the SDR may explain these results.

Notification by electronic procedures that must be validated by epidemiological surveillance unit technicians is increasing and adds opportunities for disease reporting to the surveillance system. In a study carried out in Andalusia, Spain, it was estimated that the verification process of reports received electronically accounted for approximately 10% of the activity of the personnel responsible for the surveillance of communicable diseases [26], but this not inconsiderable time is justified if, as our data suggest, it serves to increase the sensitivity of the system. Difficulties in the electronic transportability of the data generated in hospitals may explain why the sensitivity was lower for the cases reported electronically to the MRS in our study, a limitation also reported by other authors [27]. Overhage et al. [17] found that electronic data transmission from the laboratory increased paper-based reporting by more than four-fold.

This study has strengths and limitations. The main strength is that 91% of the cases reported to the two sources had reported the PIC. The detection of coincident cases (cases declared to both sources) was made through the PIC when this data was available. In cases in which the PIC was not available, the study of coincident cases was made by comparing the name and surname, age, sex, hospital or primary healthcare centre and the date of report of the case.

One limitation is that, as noted above, MRS coverage is not universal, unlike the SDR, which is. However, since both sources should be used for the monitoring and control of hepatitis A, we believe that this does not invalidate the assessment of the results obtained.

The sensitivity of the sources studied for the surveillance of hepatitis A cannot be generalized to other diseases because physicians’ perceptions of the importance of hepatitis A for public health differs from that of other diseases and because laboratory confirmation methods may be more complex for some diseases than for others [28]. In the aforementioned New Zealand study, among food-borne transmissible diseases, hepatitis A was the disease for which the reporting of cases by physicians showed the lowest sensitivity [23].

## Conclusion

In conclusion, the sensitivity of enhanced surveillance by combining two information sources (compulsory reporting by physicians and voluntary reporting by microbiology laboratories) was 59%, improving the sensitivity of each source separately and helping to improve the quality of epidemiological surveillance necessary for adequate control of hepatitis A.

The resources allocated by public health services to surveillance systems are limited, and periodic evaluation contributes to maximizing their efficiency. Capture-recapture methods may contribute knowledge of the true incidence rates of communicable diseases.
